# Thyroid Artery Embolization for Benign Thyroid Hyperplasia

**DOI:** 10.3390/jcm15072664

**Published:** 2026-04-01

**Authors:** Alan A. Sag, Saim Yilmaz, Nariman Nezami, Gary Tse, Shamar J. Young, Tim Huber, Ravi N. Srinivasa, Juan C. Camacho, Nassir Rostambeigi, Venkatesh P. Krishnasamy, Erik N. K. Cressman, Marc Sapoval, Jafar Golzarian

**Affiliations:** 1 Department of Interventional Radiology, University of Miami, Miami, FL 33136, USA; alan.sag@med.miami.edu; 2 Varisson Radiology Center, 07160 Antalya, Turkey; saimyilmaz62@gmail.com; 3 Division of Vascular and Interventional Radiology, Department of Radiology, MedStar Georgetown University Hospital, Washington, DC 20007, USA; nariman.nezami@medstar.net; 4 Harbor UCLA Medical Center, Los Angeles, CA 90502, USA; gtse@mednet.ucla.edu; 5 Department of Radiology, Vascular Interventional Radiology, University of Arizona, Tucson, AZ 85721, USA; shamar@radiology.arizona.edu; 6 Department of Vascular and Interventional Radiology, Jefferson Radiology, Hartford, CT 06106, USA; timhuber3@gmail.com; 7 Department of Radiology, Vascular Interventional Radiology, University of California Los Angeles, Los Angeles, CA 90095, USA; rsrinivasa@mednet.ucla.edu; 8 Department of Clinical Sciences, Florida State University, Sarasota, FL 34233, USA; juancamilocamacho@yahoo.com; 9 Vascular Interventional Radiology, Mallinckrodt Institute of Radiology, Washington University School of Medicine, St. Louis, MO 63110, USA; 10 Division of Interventional Radiology, University of Alabama at Birmingham, Birmingham, AL 35249, USA; vpkrishnasamy@uabmc.edu; 11 Department of Interventional Radiology, UT MD Anderson, Houston, TX 77030, USA; ecressman@mdanderson.org; 12 Assistance Publique-Hôpitaux de Paris, Hopital Européen Georges Pompidou, 75015 Paris, France; marc.sapoval@gmail.com; 13 Vascular Interventional Radiology, University of Minnesota, Minneapolis, MN 55455, USA; jafgol@gmail.com

**Keywords:** embolization, RFA, graves’ disease, thyroid nodule, thyroid, goiter

## Abstract

Thyroid artery embolization (TAE) is emerging as a potential minimally invasive therapy for benign thyroid hyperplasia (BTH), including multinodular goiter and Graves’ disease, particularly for patients who are poor surgical candidates or who decline surgery. This review summarizes contemporary evidence, technical practices, and areas of consensus and heterogeneity in this technique.

## 1. Introduction

In 2024, the American Thyroid Association (ATA) issued a statement on thyroid radiofrequency ablation [1] with input from the Society of Interventional Radiology (SIR). In this statement, the authors recognized that an emerging therapy, goiter embolization, may play a role in the non-surgical approach to patients with large benign nodular thyroid disease. A subsequent research consensus panel convened under the auspices of the Society of Interventional Radiology Foundation recognized embolization as a “safe and effective procedure with the potential to treat larger functional nodules” [2]. The largest contemporary (after 2012) publication on technique and outcomes was recently published by Yilmaz et al. [3]. Subsequent to that publication, a chapter has been published regarding anatomy and technique for Thyroid Artery Embolization (TAE) [4]. In summary, there are multiple signals internationally that embolization is emerging as a treatment option for patients with symptomatic benign thyroid hyperplasia (BTH), commonly termed “goiter”. Herein, the authors aim to succinctly summarize the state of the literature and technical trends in this emerging domain, with attention to areas of alignment and those of heterogeneity.

## 2. Disease State and the Role of Arterial Embolization

Of the nearly 100 million patients from iodine-replete areas of the world who have goiter [5], surgery will be needed in up to 15% [6], with up to a 10% reoperation rate [7] if subtotal thyroidectomy is performed to help avoid hypothyroidism. Notably, the USA maintains a 6% prevalence of goiter despite widespread iodine supplementation in the food supply [5].

Benign thyroid hyperplasia (BTH) is a cellular proliferation of the thyroid with functional (hormone-producing) or non-functional tissue. The cause is most diagnosed as idiopathic, autoimmune diseases can result in goiter by direct thyrocyte stimulation (Graves’) or by compensatory hyperplasia due to autoimmune thyrocyte destruction (Hashimoto’s). Iodine deficiency causes thyroid hyperplasia via dysregulated TSH (thyroid stimulating hormone).

A strict size criterion has not yet been established to define when a nodular thyroid becomes a “goiter”. It is generally considered to represent enlargement of the entire gland with or without multiple nodules, as opposed to solitary nodule enlargement. Most authors will concur that a thyroid size of 30 mL or greater constitutes a goiter, which is a conservative estimate for a thyroid gland that has doubled in size. Bulk symptoms attributable to goiters include dysphagia (particularly solid food dysphagia) and dyspnea (particularly supine dyspnea). These symptoms may be more pronounced once the goiter extends inferiorly through the thoracic inlet, a fixed osseous ring.

Goiters have an incidental cancer rate of 15.6% [8] (although for Graves’ goiters it is lower, 6.1%) which indicates thorough imaging and histopathologic analysis of any suspicious nodules prior to gland-sparing treatments, and has warranted surgery as the standard of care. However, not all patients are surgical candidates or willing to undergo surgery. When there is a significant retrosternal component, added surgical expertise may be necessary to create sternotomy access, which substantially increases the morbidity of the procedure. Patients are increasingly averse to large surgeries; In particular, professional vocalists and public speakers may be wary of incurring a recurrent laryngeal nerve injury in pursuit of treating a benign condition.

The main indications of TAE for BTH highlighted in the literature include:

1. Palliative monotherapy for bulk symptoms in patients refusing or ineligible for surgery. Bulk symptoms include dyspnea (particularly supine or exercise-induced), wheezing, dysphagia, or a globus sensation (sensation of persistent fullness in the throat).
2. Palliative monotherapy for hyperthyroidism. Specifically, achieving (or approaching) a euthyroid state in hyperthyroid goiter patients, particularly in the Graves’ disease population (the most common cause of hyperthyroidism) [9,10,11] where existing medical treatments carry risk for agranulocytosis, hepatoxicity, and allergic reactions over 1–2 year treatment durations with 50–60% relapse rates despite this [12,13,14,15].
3. Pre-surgical embolization. Patients with retrosternal goiter may need manubriotomy or sternotomy, increasing surgical complexity. Embolization can be considered to shrink the gland, enabling safer resection. This has been described in both the benign and malignant domains [16,17,18].

## 3. State of the Literature

Qin et al. [19] note that the first TAE animal study occurred in 1992, with Chinese-language case reports which remain underrepresented in the literature-search platforms. Around the same time, Galkin and colleagues provided one of the earliest case series of TAE for BTH, reporting treatment of 32 patients in their Russian-language publication [20] from 1994.

At the time of this report over thirty years later, a prospective study of TAE for BTH has not yet been published. Several of the largest retrospective reports are summarized in Table 1. It is important to note that safety and efficacy claims are limited by the strength of the evidence.

In the largest series of TAE for BTH, Yilmaz et al. [3] reported treatment of 56 goiters. After embolization of up to 3 thyroid arteries using 355–500 PVA mixed with papaverine, 6-month follow-up revealed goiter shrinkage from mean 147.0 mL to 62.6 mL, halving of the retrosternal extension from 31.7 mm to 15.9 mm (*p* < 0.001), and improving the quality of life from 155.4 to 70.4 (*p* < 0.001) mean score on a customized ThyPRO survey. Given this statistical significance established in a study design without a control arm, it is useful to note that the magnitude of size improvement is clinically relevant for patients undergoing consideration pre-operative embolization for goiter size reduction. In the same study, one patient developed a groin hematoma and one had symptomatic hyperthyroidism requiring management. Three patients developed transient findings (one patient with transient blurred vision resolving spontaneously within hours post-procedure, two patients with transient hoarse voice that improved with steroids). Twenty-two patients were found to have hyperthyroidism post-procedure that was subclinical. As opinion, subclinical hyperthyroidism is an expected post-embolization event.

Some studies have reported Graves’-specific outcomes. As a brief primer, Graves’ is felt to be an autoimmune disease where the patient’s B-cells (which have the unique CD-19 cell marker) create the inciting antibodies. It is thought that the culprit B-cells reside within the thyroid itself [21] consistent with findings by Zhao et al. [22] where TAE allowed partial (11%) or complete (70%) immunonormalization in Graves’ patients whereas CD-19 levels did not change post-procedure. In the same report, the ratio of T helper cells to T stimulating cells normalized in patients concordant with Graves’ immunonormalization, while natural killer cells were upregulated (which may be related to inflammatory response to the necrotic gland). Papers reporting Graves’-specific outcomes [3,22,23,24,25] appear to be concordant with each other, reporting an approximately 70–80% response rate based on different hormonal and immunohistochemical endpoints.

**Table 1:** Summary of selected published series on the topic of Goiter embolization.

Author	Description	Outcomes
Yilmaz et al. [3] 2021	Study design: Retrospective (*n* = 56) Embolic agent: PVA (355–500 micron) + Papaverine 5 mg Arteries embolized: 2 (focal nodule) or 3 (diffuse goiter)	Average shrinkage at 6 months: Focal nodules: 69% Diffuse goiter: 57% Retrosternal component: 50% Of 22 patients with non-graves hyperthyroidism, euthyroidism reached in 19 (86%)
Kaminski et al. [24] 2014	Study design: Retrospective (*n* = 22) Embolic agent: PVA (150–200 um plus 200–300 um) or Histoacryl/Lipiodol Arteries embolized: 1, 2, or 3	Total study population: normalization of thyroid function in 70.6% (study included *n* = 6 Graves’ and *n* = 16 non-Graves’)
Brzozowski et al. [23] 2012	Study design: Retrospective (*n* = 15) Embolic agent: Histoacryl/Lipiodol Arteries embolized: 1, 2, or 3	Average shrinkage at 3 months: 32% Graves’ orbitopathy improvement (2 of 2 patients) Graves’ euthyroidism reached (2 of 3 patients)
Zhao et al. [22] 2008	Study design: Retrospective (*n* = 37) Embolic agent: PVA (Details not reported) Arteries embolized: Not reported	Graves’ immunonormalization 70% Graves’ partial immunonormalization (11%) Graves’ recurrence (19%)
Xiao et al. [25] 2002	Study design: Retrospective (*n* = 22) Embolic agent: PVA (150–200 um plus 200–300 um) Arteries embolized: 3 (Bilateral superior + unilateral inferior thyroid artery)	Successful bridge to surgery in 6 patients In 16 patients, embolization was the only therapy. In that group, goiter size reduction 33–50% by 3 months

## 4. State of the Technique

Though certain published details of embolization technique vary across reports, there is broad alignment on the need for super-selection when embolizing thyroid arteries. Femoral access remains the most commonly reported approach, with emerging institution-specific interest in radial approach [26]. All patients should be heparinized as a paradigm of angiographic catheterization at the level of the aortic arch, with some institutional variations around the typical approach of 50 U/kg at the outset with additional hourly heparin injections (best practice).

There is alignment across reports on pre-embolization biopsy of the gland as a matter of best practice, although the number of biopsies is determined by local guidelines. For example while the Yilmaz series included at least one biopsy demonstrating non-malignancy (Bethesda 3), recommendations of up to two FNAs may be considered based on Korean [27], European [28], and American [1] society guidelines.

Contemporary series are aligned regarding performing subtotal gland embolization across institutions. For example, the largest reports [3,22,23,24] have focused on embolizing between 1–3 thyroid arteries at a single session. The intent of this subtotal thyroid embolization is twofold, (1) preservation of thyroid tissue to achieve/maintain a euthyroid state (and avoid daily medication supplementation) and (2) to reduce the risk of hypoparathyroidism. This is because the inferior thyroid artery (ITA) provides 80% of the blood supply to the superior parathyroid glands, and 90% of the blood supply for the inferior parathyroid glands [29]. Yet, Johansson [30] noted isolated ITA or isolated superior thyroid artery (STA) occlusion only decreased parathyroid blood flow by approximately 40% by laser-Doppler flowmetry. This implies that the known rich arterial collateral network of the thyroid [31,32,33] can supply parathyroid tissue, if at least one ITA or STA remains patent. A solitary (well-perfused) parathyroid gland is generally considered sufficient to prevent hypocalcemia. Four-vessel embolization was completed in 50% of the patients by Tartaglia et al. [34] and future studies may determine when this approach is most helpful.

## 5. Arterial Access

Thyroid artery embolization can be performed from either a femoral or radial approach [26] with a 5-F sheath depending on operator preference with certain advantages and disadvantages and also may depend on the anticipated vessels to be embolized. Yilmaz et al. [3] described unilateral superior (STA) and inferior thyroid artery (ITA) embolization for unilateral large nodules and bilateral ITA and a single STA for bilateral goiter. Operators may choose to initially only embolize the unilateral ITA or bilateral ITA for unilateral or bilateral goiter, respectively, prior to occluding the STA since the ITA reportedly supplies 80% of the ipsilateral thyroid gland [29].

Generally, for treatment of a unilateral goiter or large nodule, access to the ipsilateral inferior thyroid artery and superior thyroid artery can be achieved with a reverse curve base catheter from an ipsilateral radial approach, or from the femoral approach using a head-hunter shape catheter. Pre-operative CTA should be analyzed to determine the angulation of the thyrocervical trunk origin which helps determine optimal obliquities to see this vessel selection in profile at angiography. In the absence of detailed measurements, an ipsilateral oblique, 45–60 degree angulation of the image intensifier relative to the patient is often helpful.

For treatment for bilateral goiter, a femoral approach allows straightforward access to right and left thyroid arteries. For very torturous aortic arches, further use of stable reverse catheters can be considered such as a Simmons-1. Generally, a hydrophilic glidewire is used when manipulating across the arch vessels. Carotid catheterization requires implementation of standard neurointerventional techniques to avoid spasm, dissection, and stroke.

### 5.1. Embolic Selection and Delivery

There is alignment across recent publications on particles as the main embolic agent in TAE for BTH. For example, Xiao et al. analyzed post-embolization thyroidectomy specimens [25] showing that the average diameter of embolized capillaries was 0.12–0.25 mm. Yilmaz et al. demonstrated effectiveness for goiter size reduction using 300–500 micron particles [3] on the basis of the capillary size above. The Yilmaz technique specifies particle dilution up to 100 mL volume with a 1:1 contrast-saline ratio and inclusion of 5 mg papaverine. Smaller particles were reportedly used by Kaminsky et al. and Xiao et al., incidentally both studies focused on Graves’ patients [22,24]. Smaller size reference-ranges may be safer with the use of PVA as opposed to calibrated microspheres due to particle size properties inherent to PVA. Glue has been used [24] however there is not yet consensus on when this approach is most helpful and liquid embolics remain institution-specific for this embolic territory.

For superselection of the thyroid arteries, it is opinion that a 2.4F microcatheter is a generally accepted size allowing optimal embolization by potentially minimizing vessel vasospasm and catheter occlusion. Generally, particle sizes above 300 microns are recommended in an institution-specific fashion given prior studies showing that capillary bed in the thyroid gland may range from 120–250 microns [25]. Current best practice is to use 300–500 micron particles to avoid potential intrathyroidal arterio-arterial anastomoses. While others have used PVA with success, calibrated particles (e.g., Embospheres^®^, Merit Medical Systems, South Jordan, UT, USA) may be preferred as they are less likely to clump within the catheter or proximal arterioles. When institution-specific usage of 100–300 micron microspheres is undertaken, best practice includes caution to fully angiographically evaluate for potential shunting or collateralization to avoid nontarget embolization [18,22,23,34]. Moreover, embolization is performed with very dilute particles to further minimize potential catheter clogging which can lead to reflux and nontarget embolization if the catheter is forcefully cleared; Yilmaz et al. used 355–500 um Contour PVA diluted 1:1 with 80–100 mL volume with papaverine. Inclusion of a vasodilator in the embolic mixture is not uniformly performed.

Given the heterogeneity of literature and new emerging techniques, it is still early to recommend a single proposed pathway. For example, recent case series evaluated institution-specific pressure-enabled thyroid artery embolization (PED-TAE) using smart valved catheters to target intrathyroidal collateral vessels [35]. This technique allows for functional embolization of the superior thyroid artery without carotid access levering collaterals from the inferior thyroid artery, potentially lowering the risk of complications such as ischemic stroke or non-target embolization. Reported outcomes include thyroid volume reductions exceeding 70%. This improved efficacy may result from the use of smaller particles combined with pressure-enabled delivery. Based on data from animal and clinical liver embolization studies, PEDD is thought to enhance embolic penetration by: (1) enabling distal microvascular access, and (2) overcoming elevated intratumoral (“intra-goiter”) pressure, which limits particle delivery [36,37]. This pressure is caused by dense cellular proliferation which compresses intralesional vessels and restricts perfusion [38]. PED-TAE creates a favorable pressure gradient to improve embolic distribution within tissues [35]. A multi-center clinical trial is currently underway to further evaluate these outcomes [39].

### 5.2. Peri-Procedural Medical Management

Generally, TAE is not painful and therefore can be routinely performed under moderate sedation and even local anesthetic per institutional protocol. Intraprocedural prophylactic antibiotic choices include gram positive coverage with cefazolin.

To avoid thyroid storm, there is alignment across publications on the use of steroids as a matter of best practice in the opinion of the authors. Generally, intravenous dexamethasone (example dose, 10 mg) is given at the start of the procedure which may minimize conversion of T4 to T3 and mitigate hyperthyroid symptoms [3,22,25,35]. Pre-procedure patients with a suppressed TSH are considered hyperthyroid, denoted in the literature as “toxic” multinodular goiter. In hyperthyroid patients, if they are already on anti-thyroid drugs and beta blockers, these should be maintained without interruption in the peri-embolization period. There is less consensus across the literature regarding pre-treatment of hyperthyroid patients not already on anti-thyroid medications, though the use of thyroperoxidase inhibitor (methimazole) may be considered as a pre-treatment to inhibit thyroid hormone synthesis and disrupt iodine oxidation in the periprocedural period. Operators may choose to collaborate with endocrinology to reach or approach a euthyroid state prior to embolization, as the patient’s condition allows. Post-procedural steroids are often prescribed for several days to maintain suppression against thyroid storm. Post-operative thyroid hormone related cardiac symptoms (for example, palpitations) are managed with beta blockade. A collaborative and coordinated approach should be pre-determined in conjunction with endocrinology.

Routine shoulder or neck discomfort can be treated with NSAIDs (15–30 mg ketorolac IV) in the immediate post-procedural period, with additional acetaminophen/paracetamol and ibuprofen as needed to mitigate post embolization syndrome.

Temporary hyperthyroidism, which may be subclinical (evident on laboratory values as a suppressed TSH, but asymptomatic clinically) is expected post TAE, particularly in patients who enter the procedure hyperthyroid. Therefore, there is alignment on the need for medical management of anticipated hormone release post-embolization secondary to thyrocyte lysis. As the hyperthyroid state is transient and due to a release of pre-formed hormone, hormone-binding agents (cholestyramine) may play a more important role than medications that slow the creation of thyroid hormone (propylthiouracil, methimazole) [40] in the immediate post-embolization phase.

Biochemical follow-up should be performed post-procedure. Yilmaz et al. [3] obtained bloodwork at 2–4 day intervals until the 3-month timepoint; If patients were not hyperthyroid post-procedure this was allowed to stretch to 2–4 week intervals. In contrast, in a separate study which focused on Graves’ disease patients, Zhao et al. [22] obtained morning fasting bloodwork 3 days prior to the embolization and at 3 days, 1, 3, 6, 12, 24, 36, and 48 months following embolization. They measured T4 (total, free), T3 (total, free, and reverse), TSH, and several antibodies (thyroglobulin, thyroid microsome, thyrotropin receptor, and thyroid-stimulating).

Post-procedure cross sectional imaging has allowed reporting of goiter shrinkage, with variable reports of timing. Imaging at the 3-month [23], 6-month [3], and 12-month [24] time points have been demonstrated, though, in the presence of clinical improvement without consideration of retreatment, and outside of research protocols that would require goiter measurement to report size reduction, it is worth considering the optimal imaging schedule and modality that suits the needs of the patient.

## 6. Future Directions

The management of BTH has remained largely unchanged over the last three decades, especially compared to other endocrine organ hyperplasias such as uterine fibroids and benign prostatic hyperplasia. The application of embolization to the thyroid addresses an unmet need for gland volume reduction and hormone regulation in patients who refuse or are not candidates for medical, radioactive iodine, and surgical treatments. Adoption of this technique will depend on alignment around best practices in patient selection, technique, and patient management. Published series demonstrate a low complication rate and overall reduction of the embolized territory size by approximately 50% by 6 months (size endpoint) or an overall response rate of approximately 70–80% regarding hormonal/immunonormalization in Graves’ patients (functional endpoint). There is broad alignment on the need for pre-embolization biopsy to exclude malignancy, the use of particles as the embolic agent, and the need for a post-procedure plan to prevent, detect, and manage clinical hyperthyroidism. There is heterogeneity in the exact particle size, use of vasodilators, number of vessels embolized, biochemical/immunohistochemical laboratory values, and use of antithyroid medications which limit external generalizability of pooled data analysis of the available literature. Future work may consider combination therapy with radiofrequency ablation, as well as prospective studies with enriched cohorts (Graves’ versus non-Graves BTH; Goiter with retrosternal component) and to standardize the technical parameters toward cost-conscious, reproducible outcomes.

## Data Availability

No new data were created or analyzed in this study.
